# Supplementary material for: The PAK1-Stat3 Signaling Pathway Activates IL-6 Gene Transcription and Human Breast Cancer Stem Cell Formation
Source: Cancers (Basel). 2019 Oct 10;11(10):1527. doi: 10.3390/cancers11101527 (PMC6826853; doi:10.3390/cancers11101527)
Supplement: Supplementary file 1 [file cancers-11-01527-s001.pdf]

Supplementary Materials

Table S1. Specific primer sequence of Real-time RT-PCR.

Genes	Primers
IL-6	Forward: 5′- AGACAGCCACTCACCTCTTCAG<br>Reverse: 5′- TTCTGCCAGTGCCTCTTTGCTG
ChIP and Re-ChIP IL-6	Forward: 5′-GTTGTGTCTTGCGATGCTAAAG<br>Reverse: 5′-GCTTCTCTTTCGTTCCCGGTG
CD44	Forward: 5′-AGAAGGTGTGGGCAGAAGAA-3′<br>Reverse: 5′-AAATGCACCATTTCCTGAGA-3′
Nanog	Forward: 5′-ATGCCTCACACGGAGACTGT-3′<br>Reverse: 5′-AAGTGGGTTGTTTGCCTTTG-3′
C-myc	Forward: 5′-AATGAAAAGGCCCCCAAGGTAGTTATCC-3′<br>Reverse : 5′-AGCAAAACCCGGAGGAGT-3′
Sox2	Forward : 5′-TTGCTGCCTCTTTAAGACTAGGA-3′<br>Reverse : 5′-CTGGGGCTCAAACTTCTCTC-3′
Oct4	Forward: 5′-AGCAAAACCCGGAGGAGT-3′-3′<br>Reverse: 5′-CCACATCGGCCTGTGTATATC-3′
β-actin	Forward: 5′-TGTTACCAACTGGGACGACA-3′<br>Reverse : 5′-GGGGTGTTGAAGGTCTCAAA-3′

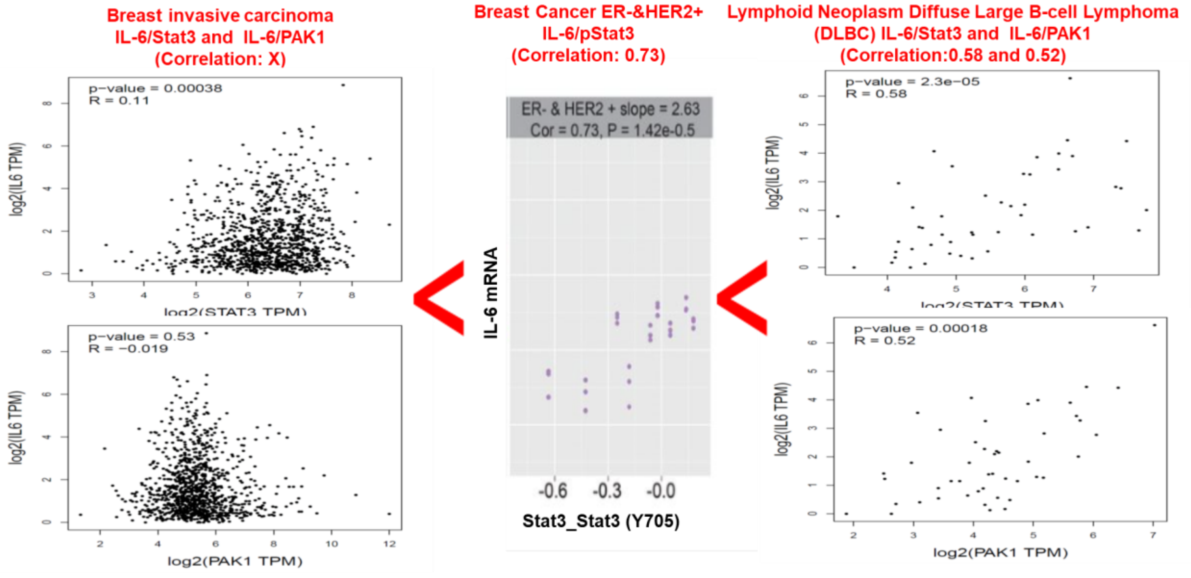

Figure S1. Correlation of Stat3/IL-6 and PAK1/IL6 in breast invasive carcinoma, breast cancer ER-&HER2+, and lymphoid neoplasm diffuse large B-cell lymphoma. Breast invasive carcinoma showed no correlation of IL-6/Stat3 and IL-6/PAK1. Breast cancer ER-&HER2+ showed that correlation of IL-6/pStat3 is 0.73. Lymphoid neoplasm diffuse large B-cell lymphoma showed that: correlation of IL-6/Stat3 and IL-6/PAK1were 0.58 and 0.52.

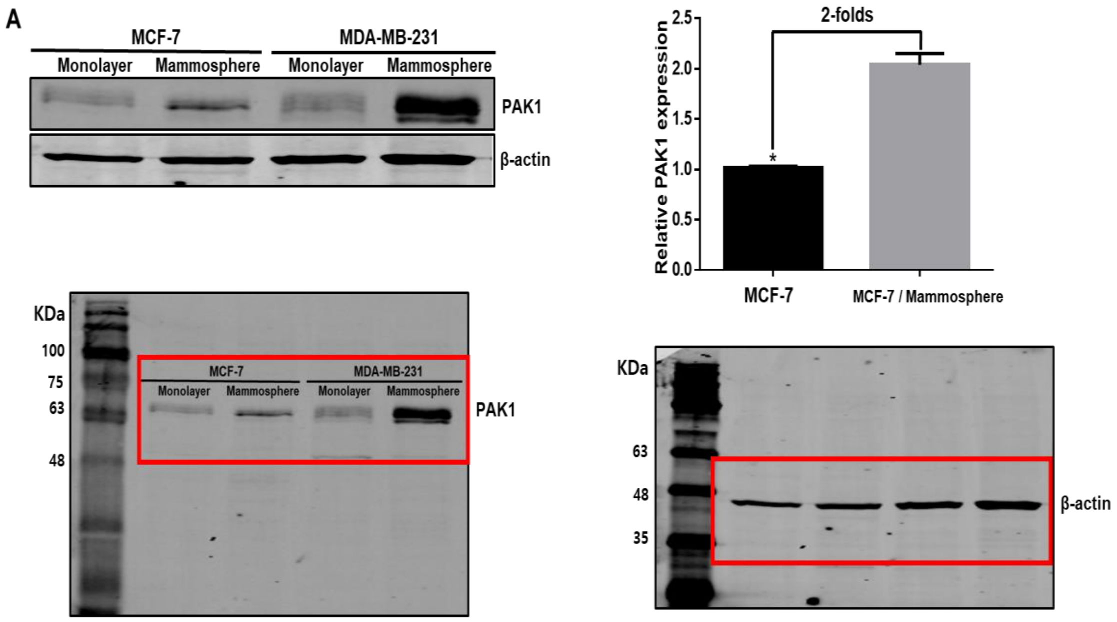

Figure 1A

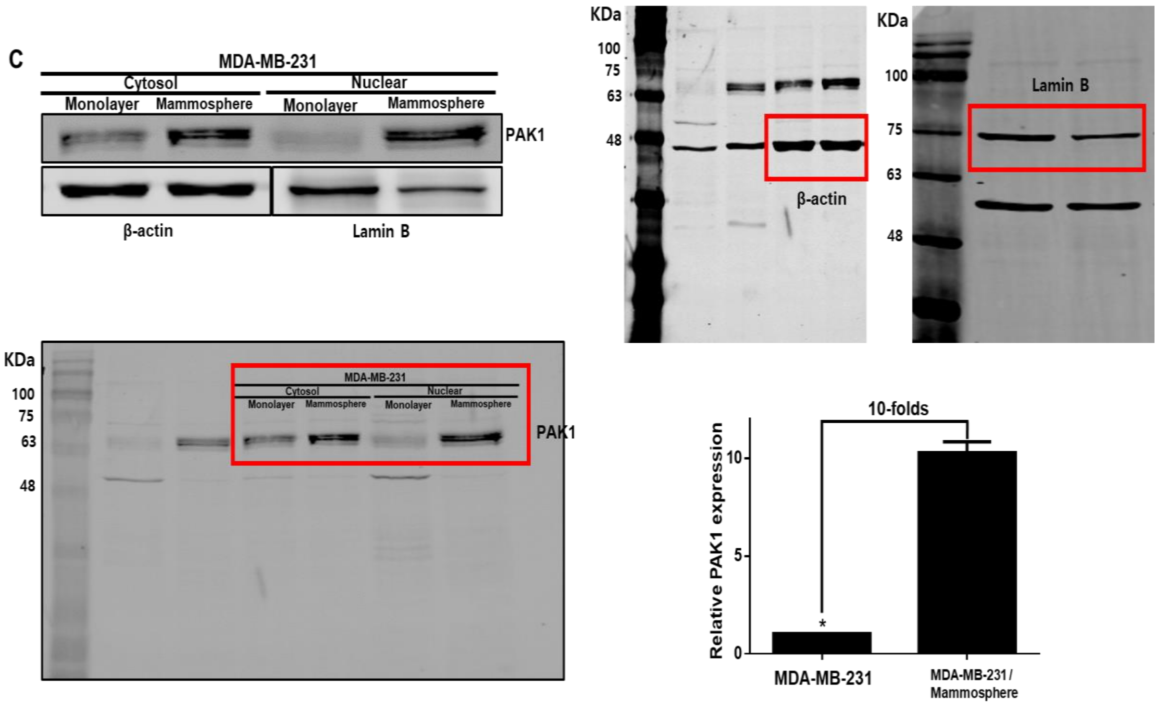

Figure 1C

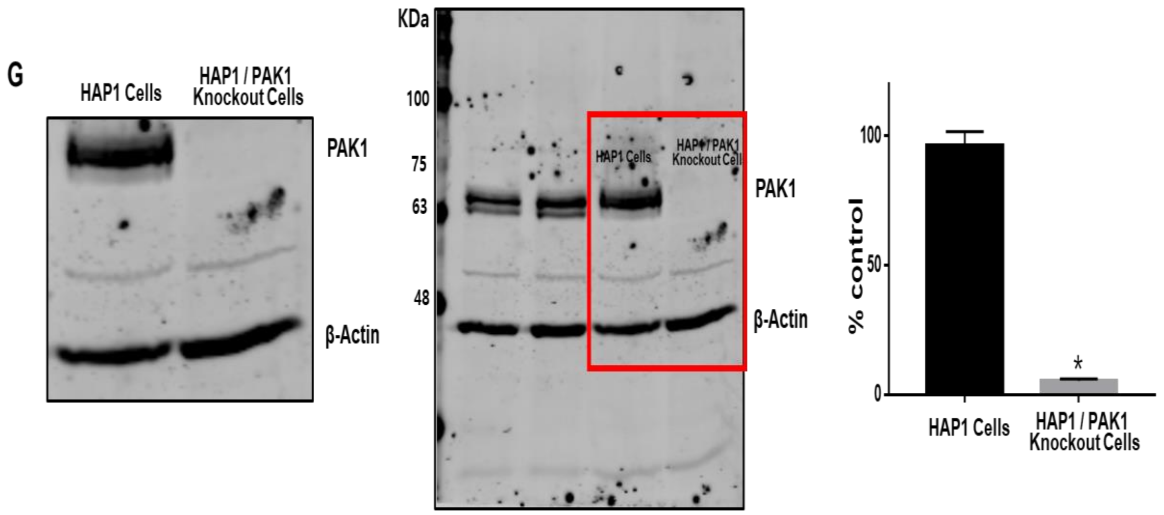

Figure 2G

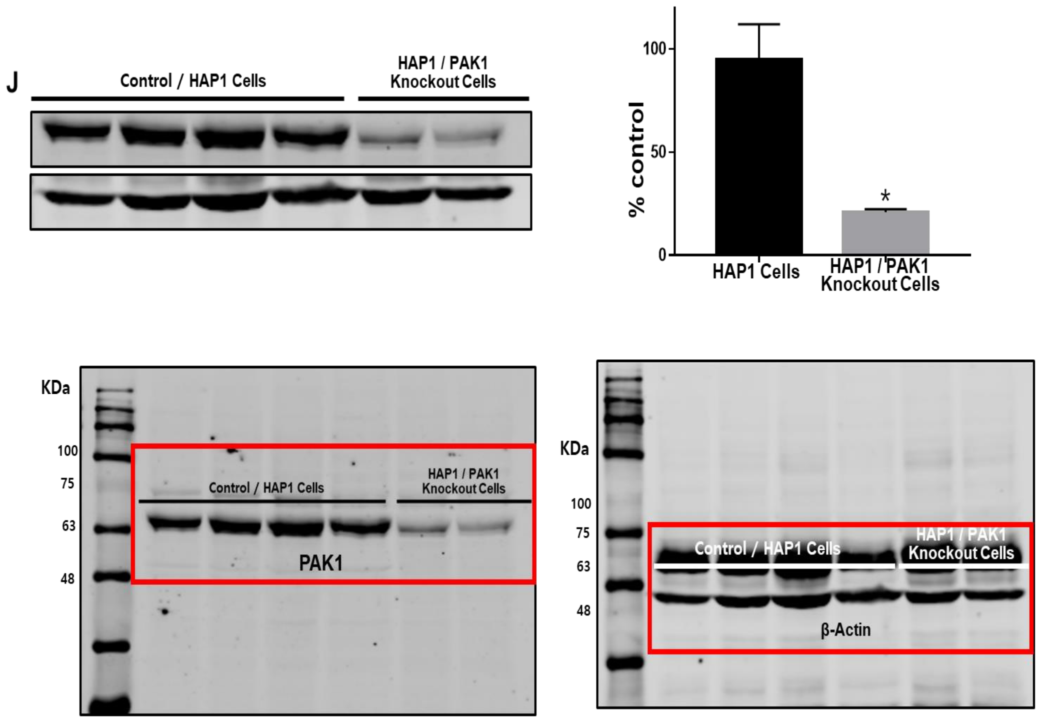

Figure 2J

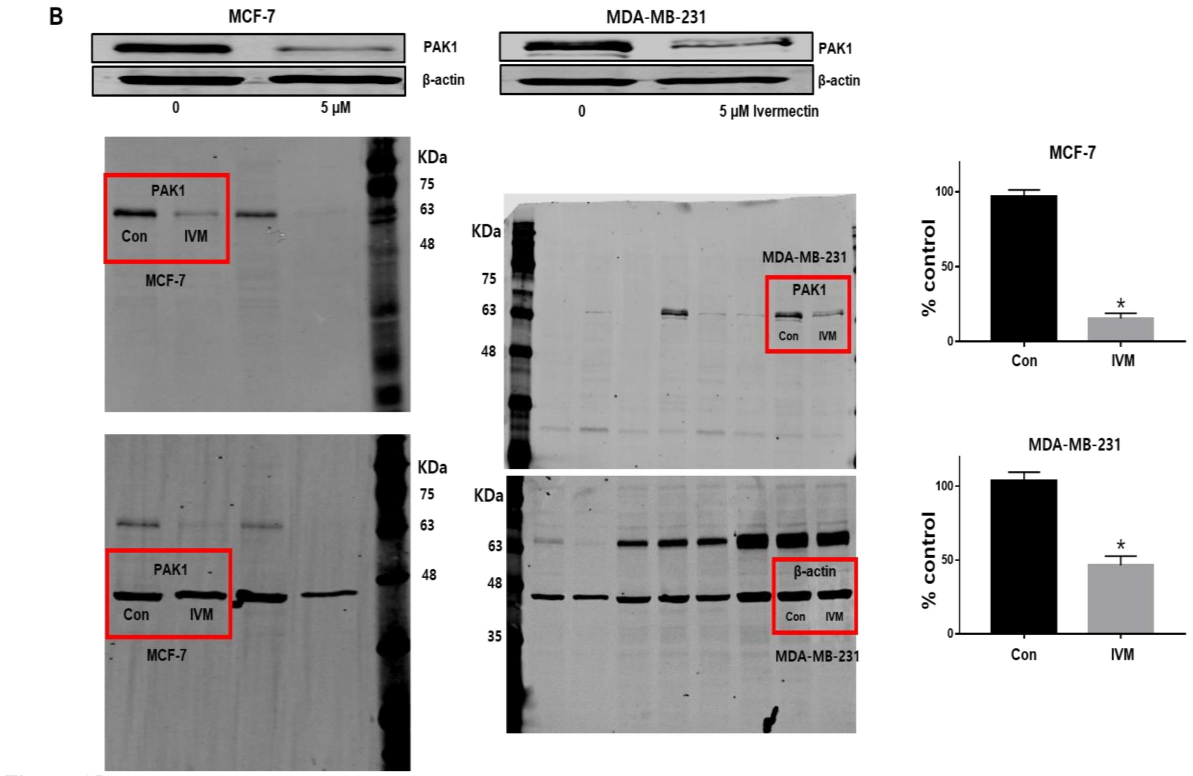

Figure 3B

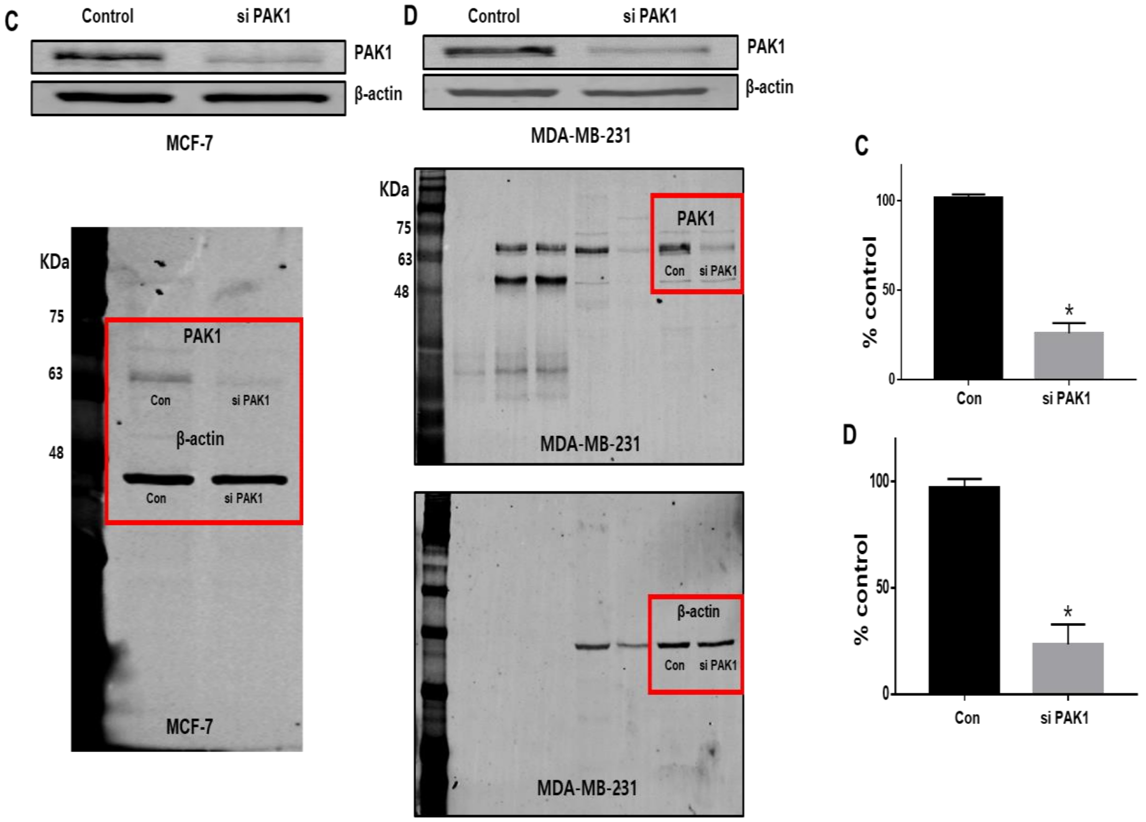

Figure 3CD

B

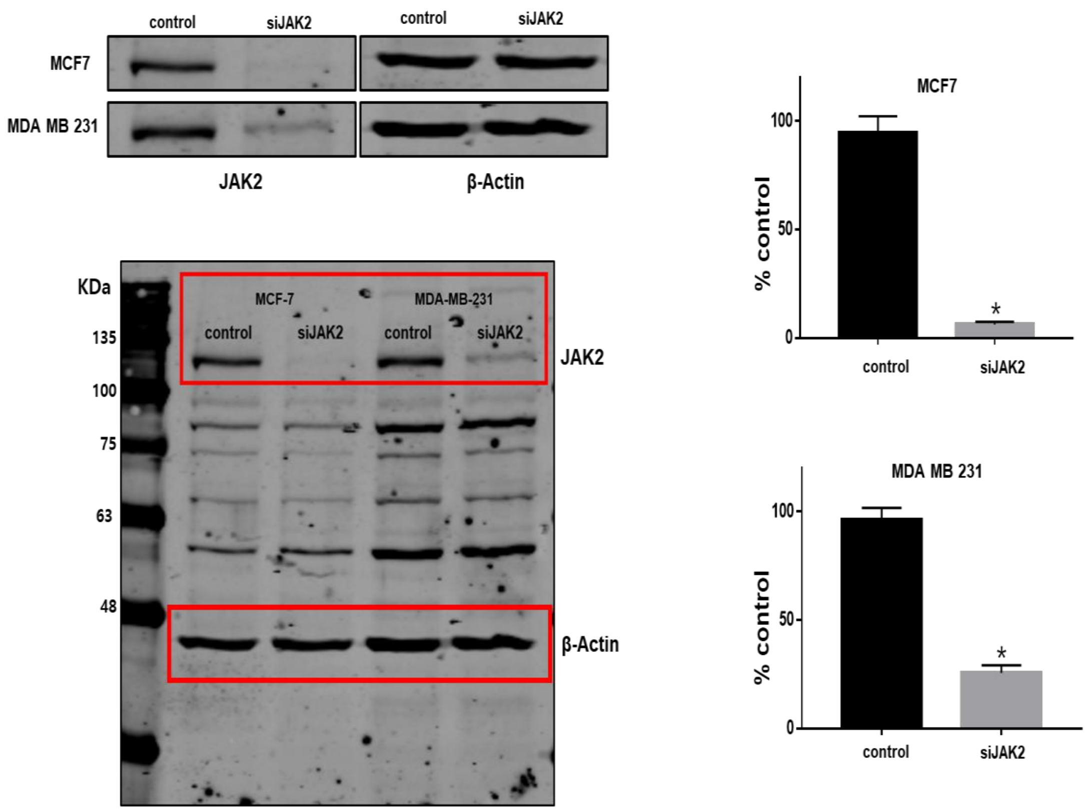

Figure 4B

E

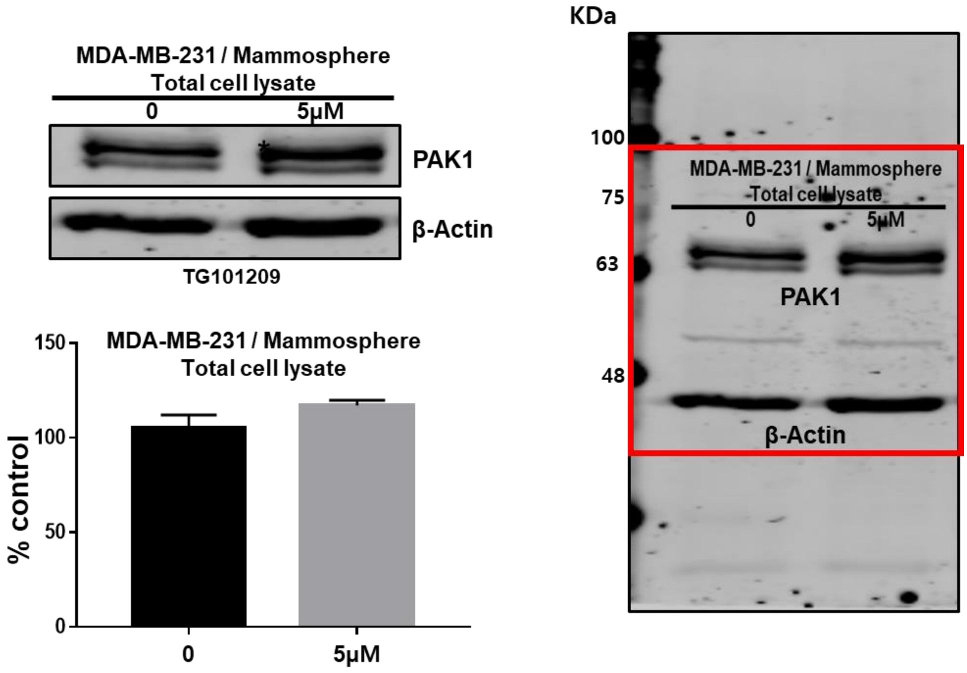

Figure 4E

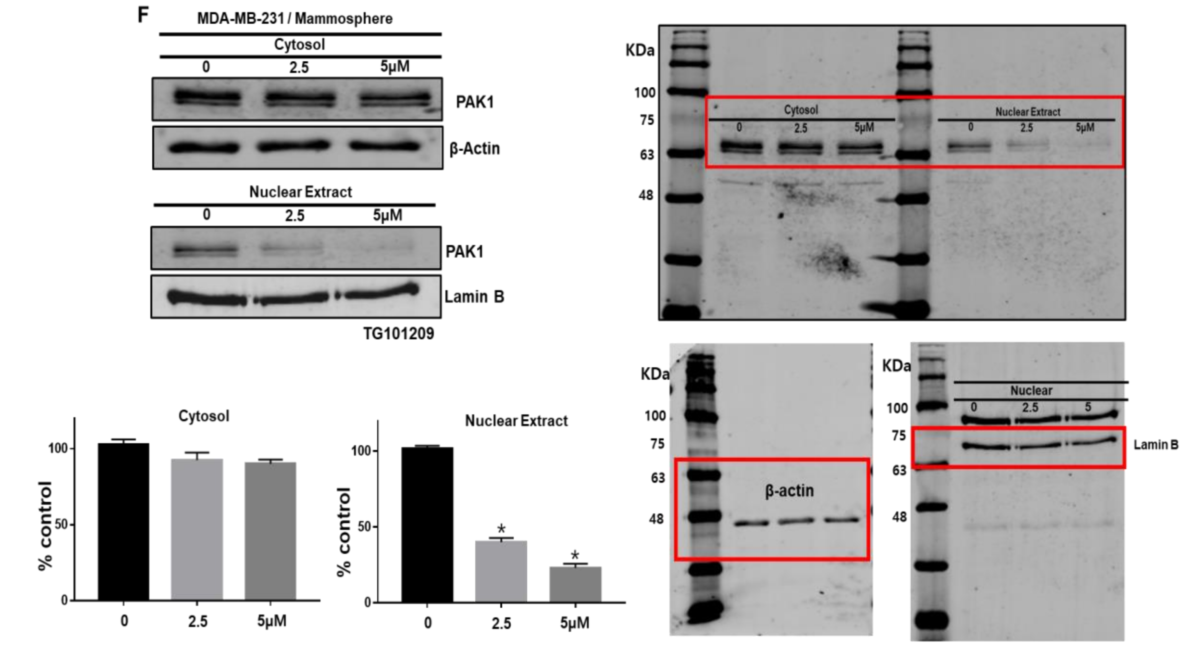

Figure 4F

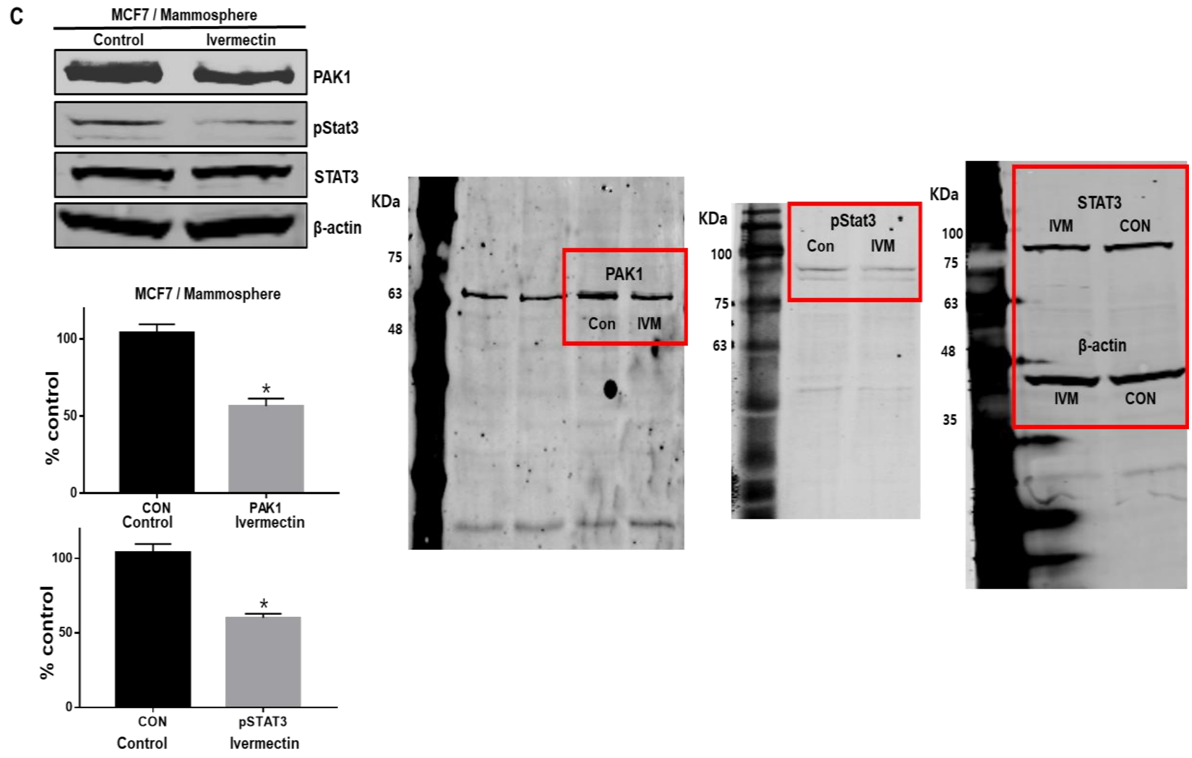

Figure 5C

C

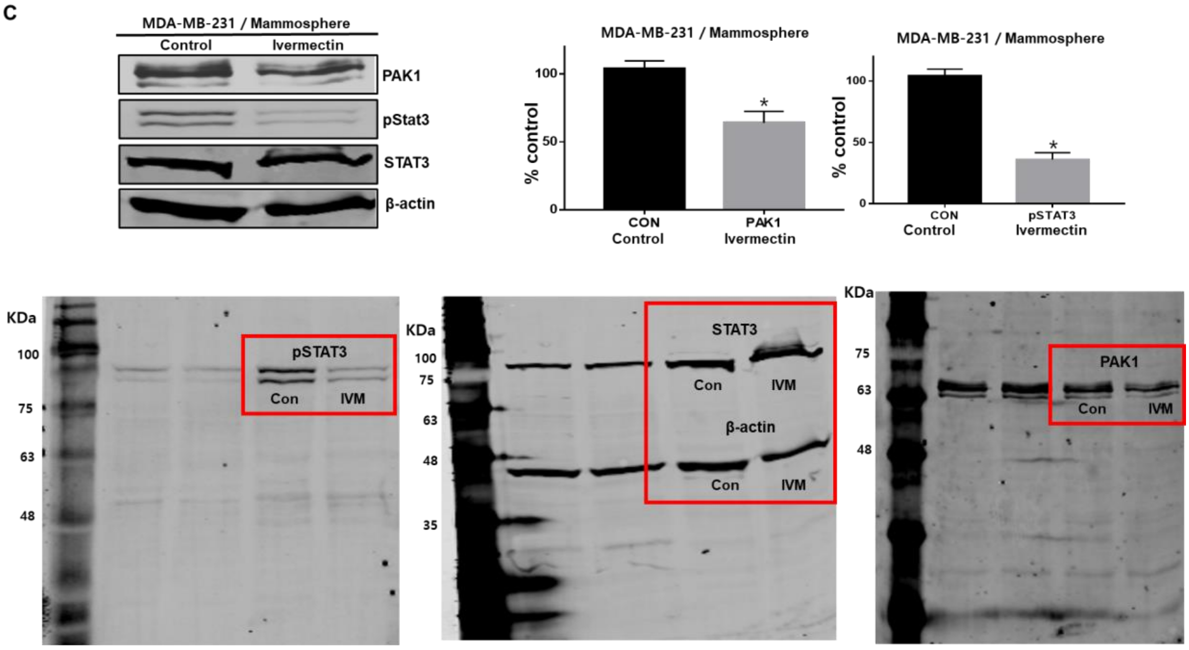

Figure 5C

B

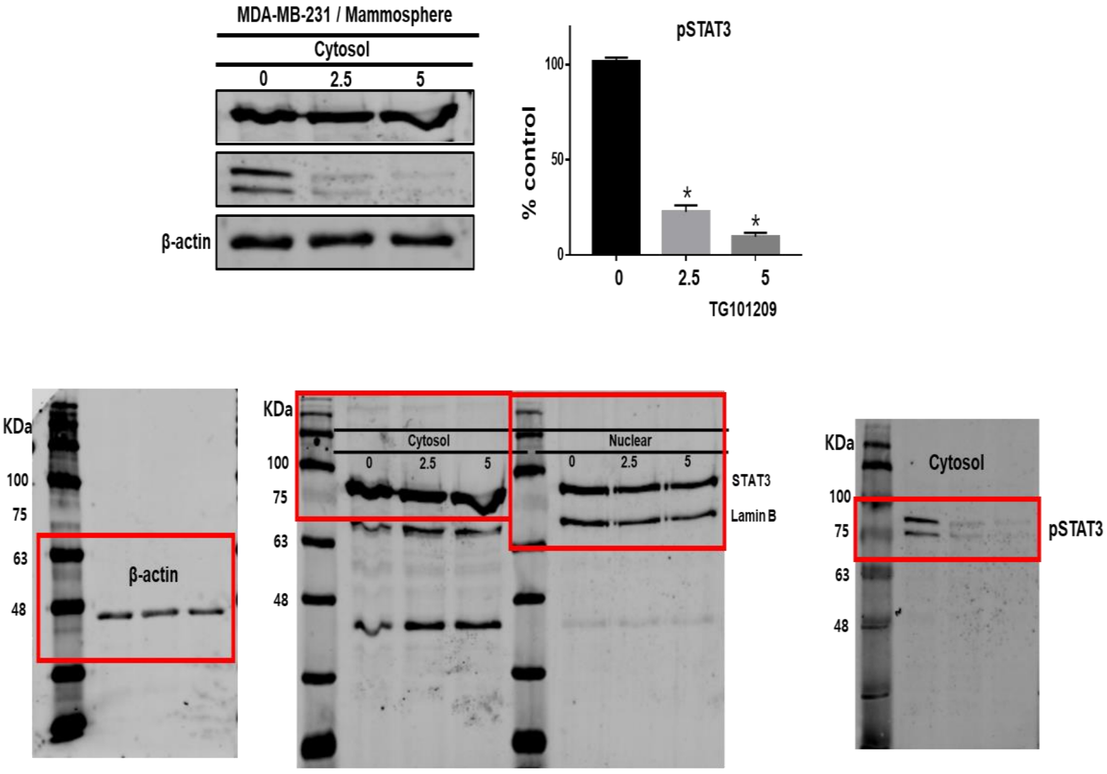

Figure 6B

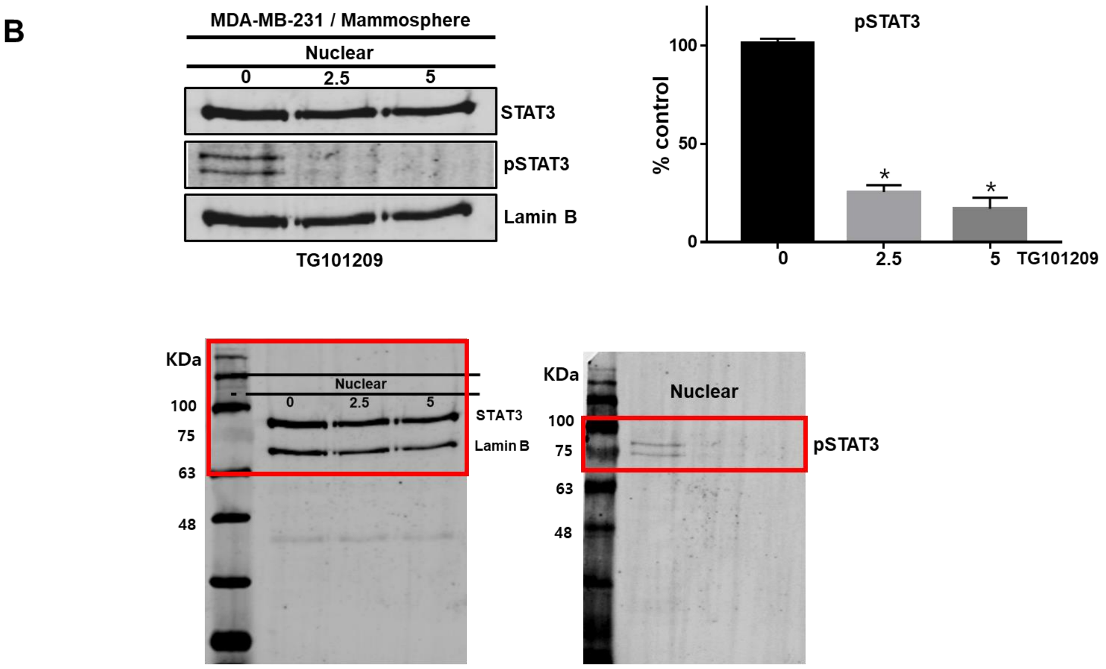

Figure 6B

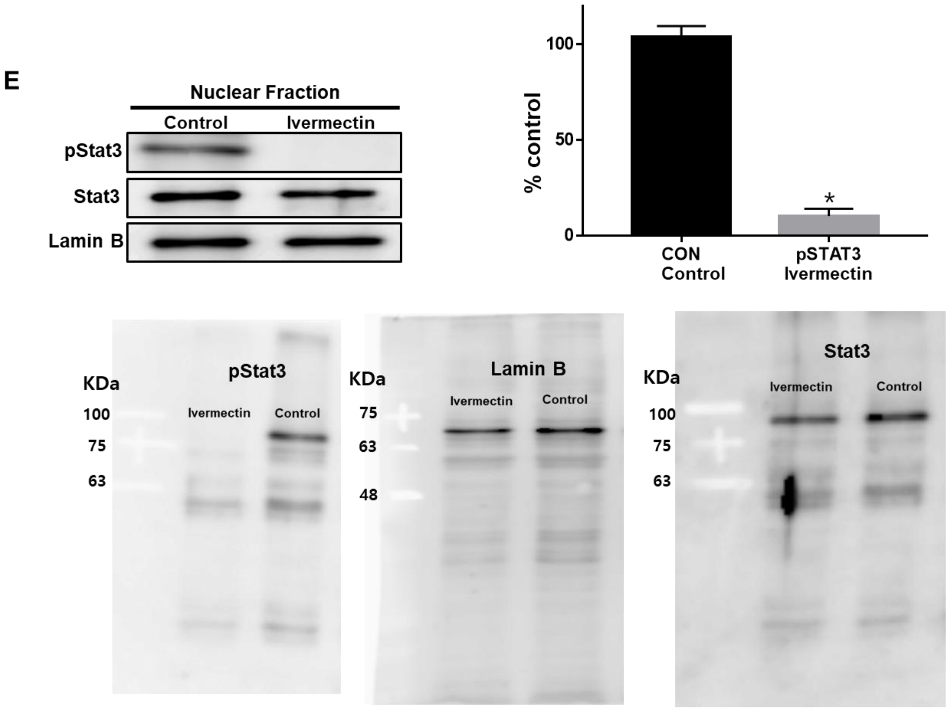

Figure 7E

Figure S2. Western blot figures including densitometry readings/intensity ratio of each band, the whole blot, and all the bands with all molecular weight markers on the Western blot.
